# The role and mechanism of gut microbiota-derived short-chain fatty in the prevention and treatment of diabetic kidney disease

**DOI:** 10.3389/fimmu.2022.1080456

**Published:** 2022-12-19

**Authors:** Pengyu Tao, Jing Ji, Qian Wang, Mengmeng Cui, Mingfeng Cao, Yuzhen Xu

**Affiliations:** ^1^ Department of Nephrology, Seventh People’s Hospital Affiliated to Shanghai University of Traditional Chinese Medicine, Shanghai, China; ^2^ Department of Endocrinology, Shanghai Municipal Hospital of Traditional Chinese Medicine, Shanghai University of Traditional Chinese Medicine, Shanghai, China; ^3^ Postdoctoral Workstation, Department of Central Laboratory, The Affiliated Taian City Central Hospital of Qingdao University, Taian, China; ^4^ Department of Rehabilitation, The Second Affiliated Hospital of Shandong First Medical University, Taian, China; ^5^ Department of Endocrinology, The Second Affiliated Hospital of Shandong First Medical University Taian, Taian, China

**Keywords:** diabetes, gut microbiota, diabetic kidney disease, pathogenesis, short chain fatty acids

## Abstract

Diabetic kidney disease (DKD), an emerging global health issue, is one of the most severe microvascular complications derived from diabetes and a primary pathology contributing to end-stage renal disease. The currently available treatment provides only symptomatic relief and has failed to delay the progression of DKD into chronic kidney disease. Recently, multiple studies have proposed a strong link between intestinal dysbiosis and the occurrence of DKD. The gut microbiota-derived short-chain fatty acids (SCFAs) capable of regulating inflammation, oxidative stress, fibrosis, and energy metabolism have been considered versatile players in the prevention and treatment of DKD. However, the underlying molecular mechanism of the intervention of the gut microbiota–kidney axis in the development of DKD still remains to be explored. This review provides insight into the contributory role of gut microbiota-derived SCFAs in DKD.

## 1 Introduction

Diabetic kidney disease (DKD) is the leading cause of death among patients diagnosed with diabetes mellitus (DM) and is also a contributor to end-stage renal disease (ESRD) (1). According to the latest data from the International Diabetes Federation, the global population diagnosed with diabetes will reach 537 million and is expected to increase to 693 million by 2045. More than 147 million people affected by DKD are from China; the disease lays a heavy social burden on the medical care system, accompanied by increased family financial costs, low quality of life, and psychological problems (2).

DKD is a complex disease often related to four main characterized pathological changes, including mesangial proliferation, glomerular basement membrane thickening, podocyte loss, and glomerular sclerosis (3). The factors contributing to these pathological changes in patients with diabetes are mainly poor control of hyperglycemia, hypertension, and micro-inflammation (4). To counteract these apparently adverse factors, the recommended first-line therapies include strict blood glucose control with a dipeptidyl peptidase-4 inhibitor or metformin, hypertension control with a renin–angiotensin–aldosterone system inhibitor, and dietary control by reduced carbohydrate intake (5, 6). However, even after following these tight controls, some patients with diabetes still develop DKD, which eventually progresses to ESRD. Thus, it is crucial to fully elucidate the mechanism for the prevention and treatment of DKD (7).

Over recent years, the research community has attached great importance to the idea of the “gut–kidney” axis, first introduced by Meijiers in 2011 (8). The increasing understanding of the “gut–kidney” axis has provided deep insight into the symbiotic relationship between the gut microbiota and the kidney (9). The human gut microbiota harbors a diverse and complex microbial community with more than 100 trillion microorganisms and serves a key role in maintaining the integrity and function of the intestinal tract, regulating immune-inflammatory responses, the absorption and metabolism of nutrients, and removing toxins (10); these functions are beneficial in maintaining a dynamic balance between the gut microbiota and the host’s health (11, 12).

Short-chain fatty acids (SCFAs), a type of saturated fatty acid containing less than six carbon atoms produced by the fermentation of the gut microbiome, are the most studied metabolite (13). The low concentration of SCFAs was observed in both patients with diabetes and diabetic mice but could be improved by supplementation (14, 15), implicating low levels of SCFAs in the pathogenesis of DKD and the positive impact of SCFA supplementation (16, 17). In this review, we summarize the functional role of SCFAs in the prevention and treatment of DKD and discuss the potential association between DKD and the intestinal microbiota microenvironment.

## 2 The pathogenesis regulating the development of diabetic kidney disease

### 2.1 The role of inflammation in diabetic kidney disease

A persistent inflammation induced by high-glucose is a leading cause of DKD *via* the activation of the generated advanced glycation end product (AGE) pathway, protein kinase C pathway, and polyol pathway, which promotes the expression of cytokines (such as monocyte-chemotactic protein-1, interleukin-1β, and toll-like receptors (TLRs) related to inflammatory pathways and macrophage infiltration leading to insulin resistance, proteinuria, and renal interstitial fibrosis (18–20). Podocytes are a type of terminally differentiated epithelial cell that play an important role in inhibiting the leakage of protein in a positive manner (21); Pyroptosis mediated by the nucleotide oligomerization domain-like receptor thermal protein domain associated protein 3 (NLRP3) inflammasome is a new type of cell death that serves as a key pathological factor contributing to accelerated DKD injury (22, 23). The absence of NLRP3 in animal disease models contributes to lowered expression of pro-inflammatory factors and improved renal fibrosis (24, 25). Studies on NLRP3 revealed suppressed activation of inflammasome and infiltration of macrophage in NLRP3 knock-out db/db mice and attenuated renal fibrosis through the blockage of the expression of profibrotic factors, such as transforming growth factor-β (TGF-β), mothers against decapentaplegic homolog 2 (Smad2), and Smad3 (26). Notably, AGEs are strongly toxic to cell survival by inducing the activation of pyroptosis (27). AGEs bind to their receptor expressed in podocytes and sharply reduce the survival rate of podocytes, leading to severe renal injury and the generation of proteinuria through the activation of NLRP3-mediated inflammation manifested by the increased expression of interleukin (IL)-1 β and IL-18 (28, 29). Other inflammatory cytokines like IL-1, IL-6, IL-18, and IL-17 with a potent pro-inflammatory impact were found to be upregulated in urine from patients with DKD, which could be employed as a potential inflammation biomarker in the diagnosis of DKD (30). In conclusion, the hyperglycemia-induced release of inflammatory cytokines is a vital indicator of DKD (31). Earlier detection and inhibition of these inflammatory cytokines may slow the development of DKD and its associated complications.

### 2.2 The role of oxidative stress in diabetic kidney disease

Oxidative stress is another key risk factor involved in the development of DKD (32). A certain amount of reactive oxygen species (ROS) is produced by metabolism in the human body and is capable of degrading bacteria (33). A normal antioxidant system can eliminate the overgeneration of ROS (34). Altogether, the balance between ROS generation and degradation by the antioxidant system plays a large part in maintaining host health (35). However, in patients with DM and poor diabetic control, hyperglycemia induces ROS overproduction that exceeds the eliminating capacity of the antioxidant system, resulting in oxidative stress (22). ROS influences the progress of DKD mainly through the dysregulation of energy metabolism, inhibition of cell growth contributing to cell cycle arrest, alteration in the synergistic or antagonistic effects of a related protein, and activation of inflammation and the immune response mediated by various signaling pathways (23, 36). In one study, tubular epithelial cells treated with high-glucose medium induced ROS over-production leading to the deposition of mesangial cells and thickening of glomerular basement membrane *via* increased expression of TGF-β and collagen-I (37); ROS also facilitated the expression of pro-inflammatory factors that cause kidney injury. The ROS-induced NLRP3 inflammasome-mediated pyroptosis is a novel pathway involved in the development of DKD (38). Under the diabetic condition, the antioxidant enzymes lose their capacity against oxidative stress by glycation, which leads to the accumulation of ROS, consequently causing severe damage to podocytes resulting in accelerated tubular injury (39). Nicotinamide adenine dinucleotide phosphate oxidase (NADPH) is widely expressed in renal tissue and is also a major source of ROS (40). The mechanism of the role of ROS in the formation of renal fibrosis can be interpreted as follows: High glucose induces the increased expression of NADPH oxidase 4 (NOX4), contributing to the overproduction of ROS, which activates the TGF-β/Smads pathway and other pro-fibrotic factors (36, 41). Moreover, ROS-induced mitochondrial damage aggravates DKD (42). The electrons leak from the respiratory chain and combine with oxygen to form superoxide, which promotes ROS production. Subsequently, ROS disturb the mitochondrion antioxidant capacity, followed by the overexpression of Bcl-2-associated X-protein and caspase-3, leading to the apoptosis of podocytes (43).

### 2.3 The role of autophagy in diabetic kidney disease

Autophagy, an adaptive system reacting to various stimuli, plays a significant role in maintaining the homeostasis of the intracellular environment *via* impaired protein degradation and recycling of these degraded materials as an energy source for cellular activity (44). Energy metabolism is believed to greatly impact the activation of autophagy. The mammalian targets of rapamycin (mTOR), adenosine monophosphate-activated protein kinase (AMPK), and sirtuins (SIRT) are the three best-known targets that regulate nutrient sensing pathways (45). In DKD, mTOR is a vital autophagy regulator and includes two complexes, the mTOR complex 1 (mTORC1) and complex 2 (mTORC2) (46). Under normal conditions, autophagy is negatively regulated by mTORC1 *via* phosphorylated unc-51-like kinase 1 (ULK1). However, the higher expression of mTORC1 is often detected in rodents with DKD, which leads to the inhibition of autophagy (47). In streptozotocin (STZ)-induced diabetic mice with hyperactivation of mTORC1, the inhibition of autophagy contributes to renal injury and proteinuria, and the pathological change is manifested by extracellular matrix accumulation and cell death that promote DKD progression to renal failure (48). Notably, the inhibition of autophagy was reversed by rapamycin (a mTORC1 inhibitor) in high-glucose-treated podocytes (49). On the other hand, AMPk promotes the activation of autophagy by sensing the AMP/ATP ratio (50). Excessive energy production reduces AMPk expression in animals with diabetes, which was rescued by treatment with an AMPk activator that alleviated kidney injury (51). Autophagy was also boosted in high-glucose cultured podocytes by the administration of an AMPk activator for apoptosis induction. Mechanistically, AMPk-mediated regulation of autophagy is related to the phosphorylation of ULK1, TSC1/2, and raptor and the inhibition of mTORC1 (52). The effect of SRIT1 is similar to that of APMk in inducing autophagy. Under the diabetic condition, SIRT1 expression is largely suppressed, inhibiting autophagy and contributing to accelerated renal damage (53). The treatment of the animal model with resveratrol (a SIRT1 activator) led to the elevated expression of SIRT1, which could restore autophagy activity and thus protect cells from hyperglycemia. Particularly, overexpression of SIRT1 in STZ-induced diabetic mice had a beneficial effect on the inhibition of podocyte damage and renal fibrosis (54). The mechanism of SIRT1-mediated protection of autophagy relies on its role in deacetylating essential autophagy proteins, such as autophagy-related (ATG)5, ATG7, and light chain3. In conclusion, the restoration of autophagy exhibited a great renal protective effect in preventing DKD (53).

### 2.4 The role of fibrotic mediators in DKD

TGF-β is key factor leading to end-stage renal disease with the features of glomerulosclerosis and renal fibrosis. Its mechanism is associated with activating intracellular signal pathways such as protein kinase and enhanced expression of cytokines to promote fibrosis, which leads to end-stage renal disease (55). Almost all types of kidney cells are capable of secreting TGF-β, and highly expressed TGF-β receptors were detected in those cell membranes. They exerted pro-fibrotic function *via* autocrine and paracrine pathways that contributed to the occurrence and development of renal fibrosis in diabetes (56). The TGF-β is an upstream regulator of TGF-β/Smads signaling pathway, which is central to the fibrotic component of diabetic kidney damage (57). Multiple exogenous stimulators such as angiotensin-II, protein kinase C, and 38 mitogen-activated protein kinase-dependent pathways could trigger the expression of TGF-β. Under diabetic conditions, hyperglycemia induced massive amounts of ROS production and attacked cell membranes, which lead to upregulation of TGF-β (58). TGF-β mediated renal fibrosis *via a* complex mechanism, including over-generation of the extracellular matrix, and dedifferentiation of tubular epithelial and glomerular endothelial cells. Smad signaling is key downstream regulator of the TGF-β pathway. In the process of renal fibrosis in an animal model with DKD, the increased level of TGF-β interacts with its receptors and triggers activation of Smad-dependent pathways, Smad2 and Smad3 presented higher expression in kidney tissues, and Smad7 was inhibited (57). Altogether, the TGF-β/Smads pathway was activated to accelerate the formation of renal fibrosis.

### 2.5 The role of abnormal metabolism regulation in DKD

The abnormal glucose metabolism in patients with DKD is characterized by the increased production of advanced glycation end products (AGEs), activation of the protein kinase C (PKC) pathway, and enhanced polyol pathways, which play a significant role in promoting the development of DKD (59). Long-term chronic hyperglycemia stimulates the overgeneration of AGEs by interacting with its receptor, which upregulated the levels of nuclear factor κB (NF-κB), vascular endothelial growth factor (VEGF), transforming growth factor-β 1(TGF-β 1), and monocyte chemoattractant protein-1 (MCP-1) (60). It was believed that progressive glomerulosclerosis in DKD is strongly associated with the increased expression of these protein levels, acting as pro-inflammatory and pro-fibrotic factors that contribute to podocyte injury and extra mesangial matrix (ECM) accumulation. Endothelial nitric oxide synthase (eNOS) is an important enzyme capable of protecting the integrity of vascular endothelial cells (61). The upregulation of PKC induced by high glucose resulted in the decreased production of eNOS and an increase in VEGF levels, which accelerated the development of DKD (62).

## 3 The role of gut microbiota dysbiosis implicated in the pathogenesis of diabetic kidney disease

### 3.1 The reduction in short-chain fatty acids generation aggravates diabetic kidney disease

Intestinal microbiota dysbiosis was observed in patients with DKD, which was attributed to the limited uptake of foods containing high fiber and fruits, resulting in the insufficient production of SCFAs (63). SCFAs, including acetate, propionate, and butyrate, are the end products of polysaccharide fermentation in the distal gut microbiome (64). The absorption of butyrate by intestinal epithelium is a major source of energy for the phosphorylation of AMPK and for promoting the release of glucagon-like peptide-1 (GLP1) (17). The acetate and propionate must bind to G-protein-coupled receptors (GPCRs)41 or 43 expressed on intestinal epithelium to perform their basic functions (17). The activation of GPR41 promotes the secretion of peptide YY (PYY) and controls satiety and intestinal transit. On the other hand, GPR43 inhibits the production of pro-inflammatory factors and enhances GLP1 secretion, which contributes to the production and induces the proliferation of pancreatic β cells and thus exerts renal protection against DKD by reducing blood glucose levels (65). Altered gut microbiota composition was detected in STZ-induced diabetic mice and led to a decrease in the concentration of SCFAs, causing a decline in the secretion of PYY and GLP1, and hence accelerating the development of DKD manifested by proteinuria, loss of renal structure integrity, and renal fibrosis (66). The administration of SCFAs or GPR41 agonists can both hamper the development of DKD by inhibiting the expansion of the high-glucose-induced mesangial cell line, the generation of ROS, and suppression of the expression of pro-inflammatory cytokines, such as monocyte-chemotactic protein-1 (MCP-1) and IL-1β (67). These aspects altogether prove that the low level of SCFAs in the intestinal tract due to intestinal dysbiosis in DM patients has a strong link to the development of DKD.

### 3.2 The role of LPS in diabetic kidney disease

Although a tight control of hyperglycemia is effective against DM and is beneficial to the delay in the progression of DKD, in one study, some patients with diabetes did not reach the stage of DKD even after poor control over hyperglycemia (68). Multiple studies have proposed the idea of alteration in the gut microbiome composition, which serves as a novel indicator of host health. A large number of toxic metabolites are generated and accumulated in patients with DKD due to the dysfunction of gut microbiota dysbiosis in degrading these toxic metabolites that consequently lead to oxidative stress and inflammation (69). In addition, the integrity of the intestinal barrier is impaired, resulting in increased permeability and thus favoring the invasion of pathogenic bacteria and their toxic metabolites (69). The circulating pathogenic bacteria or toxic metabolites in the bloodstream attack the cells and favor the expression of an inflammatory response mediated by endogenous danger signal transduction, which affects the development of DKD (70). Lipopolysaccharides (LPSs), also called endotoxin, is one of the most potent toxic metabolites and is an antigen present on the surface of Gram-negative bacteria; LPSs regulate the activation of inflammation and immune reaction, which thus leads to numerous metabolic diseases (71). The intestinal barrier loses its integrity under dysbiosis conditions, facilitating LPS influx into the systemic circulation to distal organs such as the kidney, which leads to accelerated renal impairment. Prolonged inflammation is a major contributor to DKD. TLRs, such as TLR2 and TLR4, are implicated in the pathogenesis of DKD through the stimulation of an inflammatory response. Growing evidence supports LPS-mediated inflammation in renal tissue by the activation of TLR2 and TLR4-related pathways (72). The mechanism of accelerated DKD damage is associated with the activation of the MyD88/NF-κB pathway by the binding of LPS to TLRs that contributes to increased production of pro-inflammatory factors, including tumor necrosis factor-α (TNF-α), interleukin-1(IL-1), and IL-6 (73). In TLR2 knock-out mice, low MyD88 expression is observed in renal tissue. Meanwhile, TLR2 deficiency has a positive impact on the attenuation of podocyte damage, the reduction of proteinuria, and inhibiting macrophage infiltration. The LPS–TLR4 axis is responsible for the regulation of inflammatory reaction that results in the upregulation of TGF-β, contributing to accelerated renal fibrosis (74). It is becoming increasingly apparent that targeting the LPS–TLRs–MyD88-mediated inflammatory pathway by restoring dysbiotic gut microbiome activation may be a novel therapy against DKD.

### 3.3 The role of abnormal gut microbiota in activating the immune response

The synergistic or antagonistic correlation between the gut microbiome and immune axis is necessary for maintaining host health. The immune system eliminates pathobionts and thus plays an important role in keeping the host healthy (75). A study on DKD indicated that toxic metabolites produced by an abnormal microbial community contribute to accelerated DKD damage by obstructing the capacity of the immune system to degrade toxin materials. Enhanced activation of complement 5(C5), a vital regulator in the initial stage of the immune system, was observed in db/db mice, which promoted the overexpression of inflammatory factors and activation of the TGF-β/Smads-mediated fibrotic pathway in the kidney, causing both chronic inflammation and kidney injury (76). Under the pathological condition of DKD, the immune complexes formed by pathogens and antibodies depose in the glomeruli to stimulate complement activation, which is responsible for accelerated kidney injury *via* the recruitment of immune cells (77). A large amount of C5a resides in the intestinal tract, suggesting that the altered intestinal tract microenvironment negatively affects the abundance of C5 by modifying the composition of the intestinal microbiome. The gut tract of db/db mice is characterized by a low abundance of *Proteobacteria* and *Epsilonbacteraeota*, which were restored at the phylum level after administration of the C5aR antagonist (C5aRA) (78). Meanwhile, treatment with C5aRA also rescued the decreased production of SCFAs. Over-activation of C5-induced abnormal gut microbiota was found to be responsible for reduced production of SCFAs (79). The C5-mediated STAT3 pathway participates in the development of DKD, leading to the inflammatory response. However, SCFA supplementation exerts a positive effect on the remission of DKD by inhibiting C5 expression. Also, other toxic metabolites, such as trimethylamine N-oxide derived from permeate into the circulatory system through the intestinal epithelium and accumulate in the kidney, which can be recognized by the immune system and forms an immune complex, which subsequently initiates a series of immune-mediated reactions, such as inflammation that affect the progression of DKD (80). In conclusion, an immune reaction mediated by gut microbiota dysbiosis damages the structure and function of the kidney in the DM condition.

### 3.4 Gut microbiota dysbiosis increased intestinal permeability in DKD

Under normal circumstances, the tight junctions, intestinal epithelial cell membranes, mucus secretion, and gut innate immune defensive mechanisms are the four main parts constituting a complete gut barricade defense system, which is of great importance to preclude the entry of hazardous substances or microorganisms from the intracavity to the circulation system (81); It is becoming increasingly apparent that the occurrence of DKD has a strong link to the disrupted gut barrier defense system. Amounting studies indicated the fact that under the influence of gut microbiota dysbiosis, the permeability of the gut barricade was increased in both patients with DKD and animal models due to structural and functional abnormalities of the gut tract. The intestinal dysbiosis-derived bacteria may take advantage of the leaky gut barrier to enter the bloodstream and act as pathogens or antigens, which trigger an immune reaction. ZO-1 and occluding are two important epithelial tight junction proteins keeping a tight state of the gut tract (82). It was reported that the altered gut microbiota composition in high-fat diet-fed db/db mice caused the downregulation of ZO-1 and occluding levels, which led to increased intestinal permeability and enhanced gut microbiota dysbiosis-derived pathogen absorption, thereby speeding up the deterioration of DKD through triggering tissue inflammatory responses, ROS generation and inflammatory cell infiltration (83). Notably, these unfavorable changes could be attenuated by employing antibiotic therapy. The underlying mechanism of these beneficial effects may be associated with some agents’ capacity for improving the gut barricade and reducing intestinal permeability by regulating gut microbiota and alleviating DKD-related complications (84).

### 3.5 Other toxic metabolites derived from gut microbiota aggravated the development of DKD

The human body in a healthy state can tolerate a small number of toxic products derived from gut microbiota. However, the increased production of toxic metabolites damaged the gut barricade due to the disorders of the gut environment and alteration in the composition of the gut microbiota, which led to insulin resistance, energy metabolism disorder and immune-inflammatory response in those DKD diagnosed. Trimethylamine N-oxide (TMAO) is a waste product derived from the digestion of red meat by gut microbiota, which has recently emerged as a gut microbiota-dependent metabolite linked to DKD. TMAO participates in the regulation of lipid and glucose metabolism and influences the gut, liver, kidney, and heart through a physiological connection. Multiple studies indicated that the increased concentration of TMAO in the bloodstream is positively correlated with the occurrence of atherosclerosis, thrombosis, and diabetes (85). The elevated TMAO plasma level was observed in C57BL/6J mice after chronic exposure to the provision of supplementary choline or the TMAO diet, with overexpression of collagen and tubulointerstitium ECM accumulation (86). These pathological changes contribute to progressive renal functional loss and renal fibrosis. Moreover, clinical studies have demonstrated that, compared to non-diabetes, the TMAO plasma level is higher in patients with diabetes (87). The unhealthy lifestyle and lack of fiber in the diet have become a major part of modern life and contribute to disorders of the gut microbiota, which are widely found. The uremic toxins, a waste product generated by the imbalance of gut microbiota, play a regulatory role in the activation of various cellular signaling pathways that mediate inflammation, oxidative stress, and apoptosis and influence the development of DKD (88). Under the influence of uremic conditions, the persistent activation of aryl hydrocarbon receptors (AhRs) induced by overgeneration of indoxyl sulfate (IS) results in the apoptosis of podocytes, and a decline in the glomerular filtration rate, and upregulation of pro-inflammatory cytokines (89). In addition, phenyl sulfate (PS), a metabolite derived from intestinal microflora, is reported to be associated with the occurrence of proteinuria in diabetes patients. Taken together, these systemic circulating toxic metabolite levels could be employed as a hallmark of gut microbiota dysfunction.

**Figure 1:**
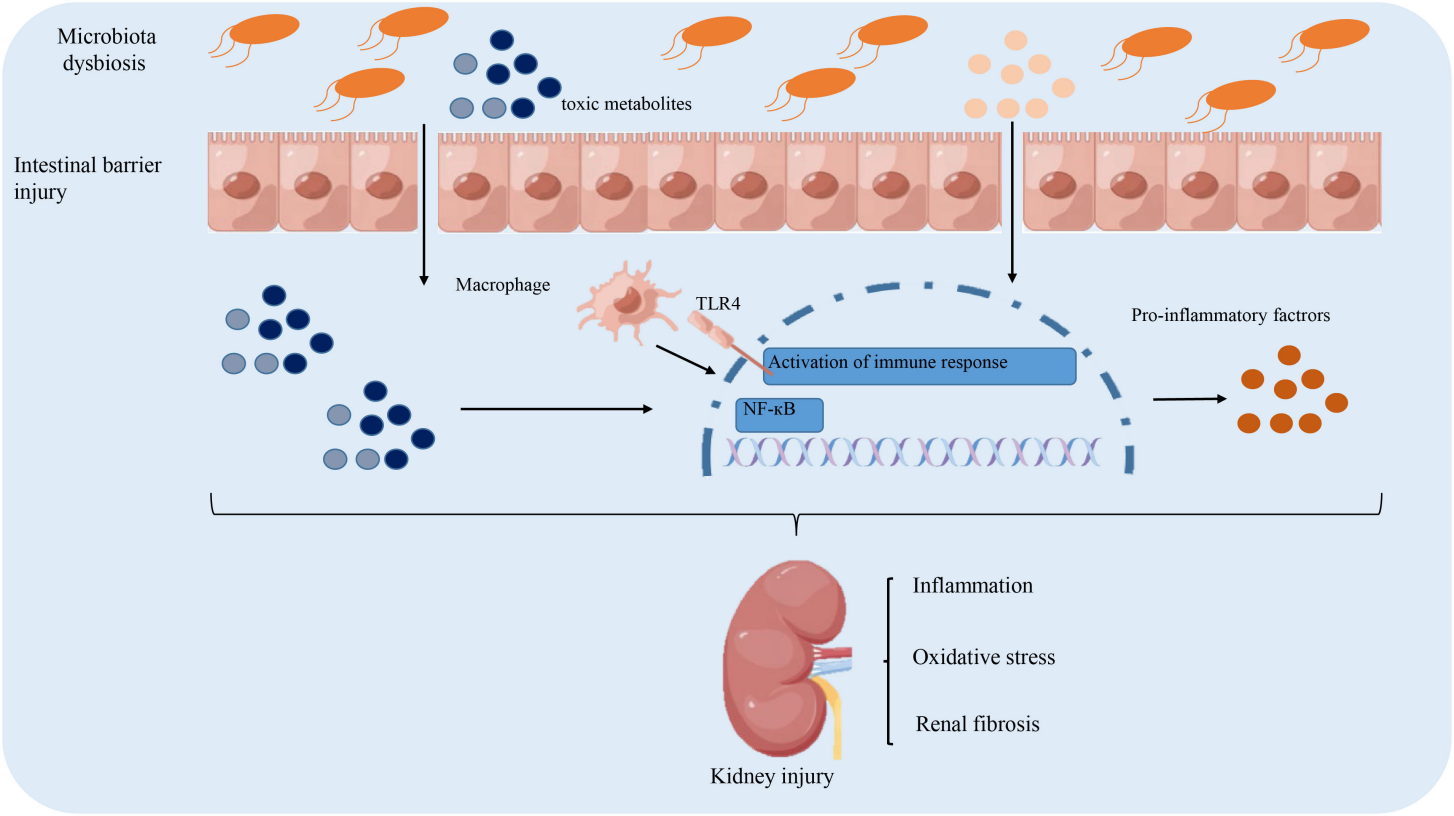
The role of gut microbiota dysbiosis implicated in the pathogenesis of diabetic kidney disease. Intestinal dysbiosis results in the accumulation of toxic metabolites, which damage the integrity of the intestinal barrier to facilitate the influx of toxic metabolites into the circulation. This triggers an immune response to secrete pro-inflammatory cytokines that contribute to renal fibrosis and oxidative stress. Altogether, these pathological factors lead to the accelerated progression of DKD.

The above-discussed mechanism of intestinal microbiota dysregulation in the pathogenesis of DKD is summarized in **Figure 1**.

## 4 The role of short-chain fatty acids in the prevention and treatment of the diabetic kidney disease

**Figure 2:**
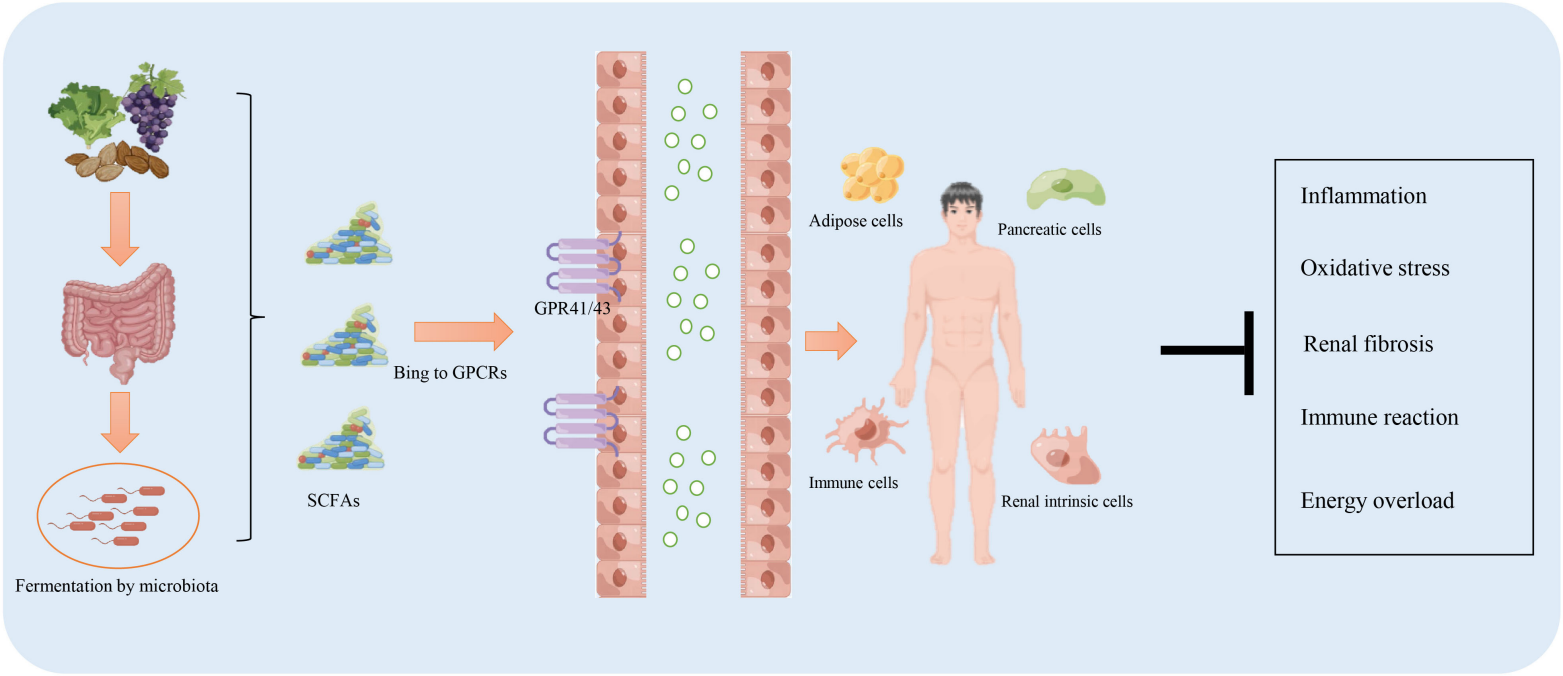
The function of short-chain fatty acids (SCFAs) in the prevention and treatment of diabetic kidney disease. The intake of a high-fiber diet promotes the production of SCFAs through fermentation by the microbiota residing in the digestive system. SCFAs facilitate resistance to inflammation, the immune response, energy overload, and renal fibrosis mainly *via* binding to G protein-coupled receptors (GPR)41/43.

Sufficient production of SCFAs ensures the host’s health by exerting multiple protective effects against various diseases, including diabetes, obesity, and cardiovascular disease. This beneficial outcome is contributed by SCFAs in regulating energy metabolism, inhibiting inflammation and oxidative stress, and regulating the immune response. Studies on the role and related mechanisms of SCFAs in the prevention and treatment of DKD are summarized in **Table 1** and **Figure 2**.

**Table 1:** Short-chain fatty acids exert multiple functions against diabetic kidney disease.

Effects of SCFAs	Protective mechanism-related index	References
Anti-inflammation	Reducing pro-inflammatory factors: IL-1, IL-6, TNF-α. Blocking the activation of the NF-κB pathway.	Wu et al. (90)
Anti-oxidative stress	Inhibiting HDAC-mediated NOX2/ROS signaling pathway to reduce ROS generation.	Al-Harbi et al. (91)
Improvement in energy metabolism	Promoting the secretion of GLP1and PYY to increase insulin sensitivity, inhibit gastric emptying, and reduce body weight.	Everard et al. (65)
Improvement in renal function	Protecting the structural integrity of podocytes to reduce proteinuria.	Li et al. (15)

TNF-α, tumor necrosis factor-alpha; IL-6, interleukin 6; NF-κB, nuclear factor kappa beta; HDAC, Histone deacetylase; NOX, NADHP oxidase; ROS, reactive oxygen species; GLP1, Glucagon-like peptide-1; PYY, peptide YY.

### 4.1 The role of short chain fatty acids in regulating energy metabolism

The SCFAs provide energy for the maintenance of normal physiological activity of the gut tract (92). The disturbance in energy supply caused by the SCFAs is attributed to the altered composition of intestinal microbiota that causes unwanted effects such as obesity, poor diabetic control, and severe DKD-related vascular diseases. To date, studies have focused on the protective effect of SCFAs as energy metabolism regulators in treating DKD (13). Low production of SCFAs was found in patients with DKD. The administration of SCFAs or transplantation of normal fecal samples from individuals with normal blood glucose could have a positive impact on reducing blood glucose and proteinuria and improving life quality (13). The dietary supplementation of SCFAs or a high fiber diet (a major source of SCFAs) could attenuate albuminuria excretion, lower blood lipids, and prevent renal glomerulus injury in STZ-induced diabetic mice. These positive results were not only observed in animal experiments but also in clinical studies on human beings. In mice with an obesity or DKD diagnosis, less progress was made in controlling the fasting blood glucose level or the body weight after strict control of food intake and drugs for therapy. However, after long-term treatment with acetate or butyrate, DKD or obesity were relieved (65). The high affinity of SCFAs for binding GPCRs is necessary for multiple functions of SCFAs. The protective mechanism of SCFAs in regulating a stable blood glucose level may be related to their involvement in glucose synthesis. GLP1 and PYY are two important hormones responsible for energy metabolism *via* SCFA-mediated GPCR activation (15). The binding of SCFAs to GPR43 expressed on the cell membrane directly stimulates the goblet cells of the colon to secrete GLP1; the generation of GLP-1 is necessary for the induction and proliferation of pancreatic β cells to perform their role in controlling body weight, reducing blood glucose level, increasing insulin sensitivity, and inhibiting risk factors associated with diabetes (67). The SCFA-mediated GPR41 pathway stimulates entero-endocrine cells to secrete more PYY, which suppresses the increase in blood glucose levels, prohibits gastric emptying, and reduces food intake. In conclusion, the SCFA-mediated GPRs pathway modulates energy metabolism by regulating the secretion of different hormones.

### 4.2 The role of short-chain fatty acids in inhibiting inflammation and oxidative stress

Prolonged inflammation is a characteristic of DKD. Although the mechanisms by which gut microbiome influences host health and renal therapy have not yet been fully explained, the role of SCFAs has been partly implicated in modulating inflammation (20). Several in-depth studies provide evidence of SCFAs serving as a protective regulator in the reduction of inflammation *via* inhibition of monocyte recruitment and chemokine production. An investigation into the pharmacological effect of SCFAs indicated a significant attenuation of renal function after sodium propionate treatment in DM patients diagnosed with DKD (93). Propionate exerts its anti-inflammatory effect by curbing the expression of inflammatory indicators, including IL-2, IL-17, IL-6, and TNF-α, and for promoting the expression of anti-inflammatory factor (IL-10) (14). Other studies also suggest the benefits of daily consumption of a certain amount of high-fiber diet in producing more SCFAs in patients with DKD. These beneficial results were also observed in other kidney diseases, such as acute kidney injury (AKI) (94). AKI in animal models is characterized by a fast decline in renal function and a high death rate. The NF-kB signaling pathway plays an irrefutable role in promoting inflammation and contributing to AKI, which could be blocked by the SCFAs (95). The anti-inflammatory effect of SCFAs is also related to their inhibitory effect on HDAC activity. Under diabetic conditions, oxidative stress and inflammation coexist and interact with each other to enhance the condition. Toll-like receptor 4 (TLR4) is a pro-inflammatory factor that is highly expressed in diabetic mice. The oxidative stress condition results in the increased expression of NOX2, which further deteriorates the antioxidant system (94). The generation of ROS and inflammation, enhanced by the TLR4/NOX2 pathway, was reversed by acetate-mediated inhibition of HDAC activity (73). Thus, a better understanding of the benefits derived from SCFAs involved in DKD will have important clinical value.

### 4.3 The role of short-chain fatty acids in regulating the immune response

Apart from the beneficial results mentioned above, SCFAs regulate immune system homeostasis and the integrity of the intestinal epithelial barrier. The close interaction between epithelial cells and immune cells establishes a solid defense system against the invasion and accumulation of lethal microorganisms (96). Intensive studies have been conducted to understand the relationship between the SCFA-mediated immune response axis and DKD. Most patients with diabetes are troubled by various infectious diseases, such as respiratory tract and urinary tract infections, caused by the decline in immunity induced by intestinal dysbiosis (97). The STZ-induced DKD mice treated with SCFAs had a renal protective effect in terms of reduced proteinuria, a decrease in blood glucose level, and attenuated renal fibrosis. These beneficial outcomes are associated with the inhibitory effect of SCFAs on the expression of TLR4 as well as blocking NF-κB pathway activation (98). Moreover, less macrophage infiltration was observed in renal tissues. The large scale of Th17 cell infiltration was observed in the animal model of DKD, which is generally recognized as a key to initiating an immune response through the secretion of IL-17A and IL17F to produce chemokines, which in turn recruit more Th17 cells, finally leading to inflammation (99). The mechanism of SCFA-mediated renal protection depends on its ability to inhibit the infiltration of Th17 cells, reduce the permeability of the disrupted intestinal barrier, and alleviate oxidative stress conditions.

### 4.4 The role of short-chain fatty acids in anti-fibrosis

The immune-inflammatory axis plays vital roles in promoting the formation of renal fibrosis, leading to ERSD. SCFAs had shown great benefits in inhibiting immune response and inflammation. The treatment with acetate and butyrate, the main effective ingredients of SCHFAs, was reported to be capable of alleviating tubule interstitial fibrosis as well as reducing ECM deposition in STZ-induced diabetic mice (100). Increasing the SCFA-producing bacteria by XOS supplementation has a positive impact on inhibiting renal fibrosis as well as the infiltration of M2 macrophages (101). HDAC activity was known to contribute to renal fibrosis. Valproic acid was capable of inhibiting HDAC activity, resulting in the reduced phosphorylation of ERK, which further inhibited the proliferation of pericytes to block Ang II-induced fibrosis (102). Under diabetic conditions, the persistent low-grade inflammation induced higher expression of transforming growth factor beta 1 (TGF-β) presented in stimulated tubular epithelial cells, which was reversed by the administration of butyrate (103). It was worth noticing that inhibiting renal fibrosis is a significant method to delay the progression of DKD to ESRD. The mechanism of SCFAs conferring anti-fibrosis protection on DKD is mainly through inhibiting the activation TGF-β pathway, HDAC activity, and reducing phosphorylation of ERK. It is still necessary to explore other SCFAs’ related functions against renal fibrosis.

## 5 Medical therapies against gut microbiota disorders in DKD

### 5.1 The role of dietary fiber in regulating gut microbiota

Dietary fiber (DF) provides a large amount of fermentable substrate for intestinal microbiota and regulates the health of the host in an indirect manner, which is recognized as a promising method to delay the progression of DKD (104). Because of the absence of DF-degrading enzymes in the human body, DF cannot be digested and absorbed in the human body. Given its specific chemical composition and structure, DF exerts specific physiological functions, including reducing postprandial blood glucose levels, delaying gastric emptying, and altering gut microbiota composition (105). The undigested DF can be utilized as fuel for the growth of intestinal microorganisms that lead to the proliferation of intestinal flora and production of SCFA (acetate, propionate, and butyrate) through the fermentation process, and increase the abundance of Bifidobacterium and Lactobacillus (106). These beneficial results of DF contribute to anti-inflammation, anti-oxidation, and anti-immune reactions. Non-obese diabetes (NOD) mice were generally used as an animal model to study type I diabetes. After 24 weeks of treatment with long-chain or short-chain inulin fructans, the blood glucose level was significantly improved in NOD mice *via* an increased abundance of rumen Coccidae and lactic acid bacteria and an upregulated ratio of Firmicutes and Bacteroides. The intake of long-chain or short-chain inulin fructans also produced positive results in decreasing the permeability of the gut barricade and restoring the homeostasis state of intestinal microbiota (107).

### 5.2 The role of exercise in regulating gut microbiota

Lack of exercise, obesity, and stress are the major causes leading to the rising prevalence of DM. It is estimated that about 27% of DM, 30% of ischemic heart disease, and 21%–25% of breast cancer can be attributed to a lack of physical activity (108). Relevant medical guidelines suggest that a healthy diet and regular physical activity are strongly recommended as the most economical means of preventing and treating diabetes (109). Long-term (12 months) moderate intensity aerobic exercise is capable of reducing the expression of inflammatory-related cytokines, such as IL-1 β and TNF-α, with the upregulation of anti-inflammation factors like IL-4 (110). Moderate physical activity also produces a positive outcome in controlling the development of DM by promoting the secretion of GLP-1, which is helpful to improve glucose metabolism (111). After diabetes mice went through six weeks of exercise, favorable changes were observed in the composition of rat intestinal flora, including a reduction in the abundance of Bacteroides and an increase in the abundance of Firmicutes and Proteus, which promoted the generation of SCFAs (112). These SCFAs induced the secretion of peptide (PYY) limiting the intake of food and GLP-1 increasing insulin sensitivity (113). A sedentary lifestyle has a negative influence on host health because of the reduction in abundance of probiotics and the rising ratio of gram-negative bacteria, which resulted in disorders of the gut environment.

Compared with the less active group, the level of bacterial endotoxin was lower in well-trained athletes, accompanied by a higher concentration of heat shock protein, which contributed to reducing intestinal permeability *via* enhancing the upregulated tight junction proteins (114).

### 5.3 The role of probiotics, synbiotics, and postbiotics in the regulation of the gut microbiota

Probiotics are a series of indispensable living bacteria or beneficial microorganisms for human health that participate in the synthesis of various vitamins, the regulation of food digestion, the inhibition of the proliferation of pathogenic bacteria, and the degradation of toxic substances. A survey of 340 DKD patients receiving probiotic intervention had shown that the administration of probiotics exerted beneficial effects on reducing the expression of genes that are responsible for the generation of inflammation and oxidative stress biomarkers *via* significantly downregulated levels of CRP and MDA plus enhancing GSH expression (115). It was reported that the benefits of supplemental probiotics may also help to protect the gut barrier. The protective mechanism is associated with enhanced levels of occluding and claudin-1, leading to reduced intestinal permeability (116). Synbiotics, a compound consisting of prebiotics and probiotics, possess good biological functions for the prevention of gut microbiota dysbiosis. A study revealed that the intake of synbiotics containing *Clostridium butyricum* and corn bran played a positive regulatory role in reducing the abundance of pathogens while enhancing the growth of SCFA-producing bacteria, which further led to an increase in acetate and isovalerate (117). Postbiotics is an inanimate microorganism that confers a health benefit on the host. The postbiotic intervention also possesses a regulatory function in inhibiting the differentiation of immature cells into mature adipocytes, reducing body weight gain and lipid accumulation in mice by promoting the activation of the TLR2–AMPK pathway (118). Further studies revealed that the intake of postbiotics could attenuate insulin resistance as well as inhibit inflammation *via* interacting with NOD2 (119). In short, the renal protective mechanism of probiotics, synbiotics, and postbiotics is mainly associated with the promotion of the growth of beneficial bacterial metabolites (such as acetate, propionate, and butyrate) as well as protecting the gut barrier *via* limiting the production of LPS and TMAO. The intake of probiotics, synbiotics, and postbiotics also helped to suppress the activation of signaling pathways that were responsible for oxidative stress, inflammation, and insulin resistance.

## 6 Conclusion and prospect

DKD is a global health problem with a complex mechanism of pathogenesis. A full understanding of the mechanisms underlying DKD is of great significance for laying out an effective treatment plan for the disease. Inflammation, oxidative stress, and autophagy play negative roles in accelerating the progress of DKD. Treatments involving inhibition of inflammation, reducing oxidative stress, and restoring autophagy activation are shown to protect podocytes and decrease proteinuria. Recent progress in the knowledge of the “gut–kidney” axis is a novel perspective for elucidating the relationship between gut microbiota and DKD. SCFAs are beneficial end products derived from gut bacteria, which regulate a variety of host physiological functions. Nonetheless, the mechanism of attenuation of DKD-related complications by the administration of SCFAs is still in its infancy. The beneficial effects of SCFAs may be associated with their ability to regulate energy metabolism, reduce the inflammatory response, and suppress immune effects. It is apparent that the beneficial properties of SCFAs act as a promising biomarker to diagnose and treat DKD. Still, a few questions need to be addressed before the clinical application of SCFAs.

First, the binding of SCFAs to GPCRs is necessary for their diverse functions. But there are still many unknown receptors with GPCR-like effects yet to be discovered. Discovering these not-yet-known receptors and developing specific targeted drugs will contribute to solving the puzzle of DKD. Second, most of these SCFA-induced beneficial results were observed in animal experiments and have rarely been performed in humans. Further research should be conducted to confirm whether these benefits could be repeated in humans. Lastly, SCFAs undoubtedly have an irrefutable role in attenuating DKD; any possibility that other metabolites would produce the same beneficial results needs to be investigated.

We are confident that these difficulties will and must be overcome to elucidate the detailed mechanism of the function of SCFAs and their promising medical value to mankind.

## Author contributions

All authors listed have made a substantial, direct, and intellectual contribution to the work, and approved it for publication.
